# An evaluation of the structure and process of stroke rehabilitation in primary, secondary and tertiary hospitals in Ghana

**DOI:** 10.4102/sajp.v78i1.1637

**Published:** 2022-05-23

**Authors:** Tawagidu Mohammed, Gifty G. Nyante, Diphale J. Mothabeng

**Affiliations:** 1 Department of Physiotherapy, School of Healthcare Sciences, University of Pretoria, Pretoria, South Africa; 2 Department of Physiotherapy, School of Biomedical and Allied Health Sciences, University of Ghana, Accra, Ghana

**Keywords:** stroke, rehabilitation, healthcare professionals, structure, process

## Abstract

**Background:**

Evidence shows that quality indicators such as the structure and process of stroke rehabilitation can influence patient outcomes. However, not much attention has been paid to the study of these issues in low- and middle-income countries such as Ghana.

**Objectives:**

Our study evaluated the structure and process of stroke rehabilitation in primary, secondary and tertiary hospitals in the Greater Accra Region of Ghana.

**Method:**

A cross-sectional survey was conducted involving 111 healthcare professionals. The World Health Organization (WHO) situational analysis and Measure of Processes of Care for Service Providers for Adults (MPOC-SP[A]) questionnaires were administered to gather information on the structure and process of stroke rehabilitation. Descriptive statistics were used to summarise data, and chi-square and Kruskal–Wallis tests were used to establish associations and comparisons, respectively.

**Results:**

A stroke unit was only available in the tertiary hospital. Although all three hospitals had a multidisciplinary team approach to care, the constituents differed. Length of hospital-stay, duration of treatment and basis for discharge from acute care were not associated with the hospitals. Therapy sessions, access to computed tomography (CT) and magnetic resonance imaging (MRI) scanning were dependent on the hospitals.

**Conclusion:**

The structure and process of stroke rehabilitation across the three hospitals were similar in some constructs and different in others.

**Clinical implications:**

Data gathered will help to provide information on the available structure and processes of stroke rehabilitation, which could help assess the quality of care provided.

## Introduction

Stroke is the leading cause of adult disability and remains the third most common cause of death worldwide (George & Steinberg 2015; Urimubenshi et al. 2018). The long-term physical consequences of stroke create a huge burden on stroke survivors, their families, healthcare systems and the economy (Brewer et al. 2013). In addition, the social and economic impact of stroke makes it a major public health concern around the globe (Moskowitz, Lo & Iadecola 2010). It is estimated that by 2030, low- and middle-income countries such as Ghana will contribute about 80% to the global stroke caseload (Sanya et al. 2015). Currently, stroke is identified as a major cause of hospital admissions in Ghana (Agyei-Mensah & De-Graft Aikins 2010; Gould et al. 2011; Maredza, Bertram & Tollman 2015).

Stroke rehabilitation is a key component of stroke management and is reported to produce several benefits, including improvement in function and quality of life for the stroke survivor (Teasell et al. 2009). Despite the increased awareness of the benefits of stroke rehabilitation, research on this topic remains limited, particularly in low-resource countries, such as Ghana. Consequently, clinicians, administrators and policymakers struggle to develop evidence-informed policies and interventions to improve stroke services in the country (Baatiema et al. 2017a). In order to achieve the best of outcomes following stroke rehabilitation, high-quality care is needed (Hinchey 2003). However, in order to implement quality institution-based stroke rehabilitation, it is essential to identify critical aspects of stroke rehabilitation (e.g. structure and process) that may be in need of improvement in low-resource settings (Hoenig et al. 2002; Miura et al. 2019).

One of the popular models that has been extensively studied and widely used in assessing the quality of health care is the Donabedian’s structure, process and outcome model (Donabedian 2003). The structure and process parts of this model served as a guide in our study. The structure of care represents the physical and organisational aspects of the healthcare settings, in which services are provided and may include the systemic organisation, staffing, policies, protocols and equipment (Haj, Lamrini & Rais 2013). Processes involve specific activities or tasks that are implemented by health professionals to improve patient outcomes. Processes may include therapies and procedures aimed at promoting functional recovery and patient satisfaction (McNaughton et al. 2003), as well as patient education, procedural interventions and coordination of care (Donabedian 2003). Ameh et al. (2017) suggested that a good structure can influence processes, which may, in turn, produce good outcomes.

Various studies have assessed aspects of structure and process of stroke rehabilitation in high-income countries (Hoenig et al. 2002; Stuart et al. 2005). Hoenig et al. (2002) assessed aspects of structure and process including systemic organisation, staffing, technology and compliance with the Agency for Healthcare Policy and Research Stroke Rehabilitation guidelines. They found that improving the structure of stroke care might enhance the process of care for patients with stroke, which may improve the outcomes for patients. Stuart et al. (2005) also assessed multidisciplinary team coordination, baseline assessments, goal settings, treatment plan, progress, management of impairments and disabilities, prevention of complications and recurrent stroke, family involvement, patient and family education, and discharge planning. They concluded that improvements in functional outcomes depend on optimal timing, duration, intensity and setting for stroke rehabilitation. However, there are still insufficient data on the structure and process of stroke rehabilitation in low-income counties such as Ghana. A systematic review by Akinyemi and Adeniji (2018) found that data on stroke services in Africa are limited and the uptake of dedicated stroke units remains limited. As rehabilitation is diverse in the settings in which it is provided, it is therefore important to find out what is available for stroke rehabilitation services in low-income countries such as Ghana.

Knowledge of the existing structure and process of stroke rehabilitation may provide insights to support political and financial investments in stroke rehabilitation in low-income countries such as Ghana. As little is known about the structure and process of stroke rehabilitation in low-income countries, a better understanding of current practices would be of value in facilitating interventions to improve the quality of stroke rehabilitation in Ghana.

The aim of our study was to evaluate the current structure and processes of stroke rehabilitation in the Greater Accra region of Ghana. The following objectives were formulated:

- to evaluate the current structure of stroke rehabilitation at three selected hospitals in the Greater Accra region
- to assess the processes of stroke rehabilitation available at the three selected hospitals in the Greater Accra region.

## Method

Our cross-sectional survey was carried out in the medical wards and stroke unit of a primary hospital (Amasaman District Hospital), secondary hospital (Tema General Hospital) and tertiary hospital (Korle Bu Teaching Hospital) located within the Greater Accra region of Ghana. These hospitals were selected as they represent the three healthcare delivery systems in Ghana and are all involved in acute stroke rehabilitation. A total of 111 (*n* = 111) healthcare professionals (HCPs), including medical personnel, nurses, physiotherapists, occupational therapists, speech and language therapists, clinical psychologists and dieticians, were recruited by convenience sampling. The participants were included if they had worked in stroke rehabilitation for at least one year and if they were specialists in stroke rehabilitation.

A total of 159 questionnaires were initially distributed, of which 32 were not returned and 16 rejected. Therefore, the minimum number of participants was obtained using the Cochrane formula; *n* ˃ Z*2* (*P*) (1–P) / E*2* (Cochrane 1977). At a confidence interval of 95% and standard score (*Z*) of 1.96, population proportion (*P*) of 0.5, with an allowable error (*e*) of 0.093, a minimum of 111 HCPs were included. Half the quota for participation was assigned to Korle Bu Teaching Hospital as it is the largest hospital in Ghana and has the largest number of staff.

### Data collection instruments

A self-designed information sheet was used to gather information about the age and gender of participants. Two questionnaires were administered to gather information on the structure and processes of stroke rehabilitation.

The World Health Organization’s (WHO) questionnaire for situational analysis was used to gather information on the current structure and processes of stroke rehabilitation available in the selected hospitals. Elements of structure that were assessed included rehabilitation units, staffing, management protocol, payment system, referral system and assistive devices. Elements of process that were evaluated included rehabilitation sessions and duration, Computed tomography or magnetic resonance imaging (CT or MRI) scan availability, length of hospital stay, basis of discharge and discharge destinations. This questionnaire was used by Christian et al. (2016) for evaluating rehabilitation capacity in the Ghanaian population. It has also been validated in Ghana (Osen et al. 2011). It is a self-administered questionnaire and takes about 15-20 min to complete. The questionnaire is closed-ended and was adopted with modifications to fit the objectives of our study. The questionnaire was validated by an expert in stroke rehabilitation, and then piloted and analysed by the first author to assess the response latency and to ensure clarity before adopting it for our study.

The Measure of Processes of Care for Service Providers for Adults (MPOC-SP[A]) questionnaire was used to assess the implementation of family and patient-centredness of care in the process of stroke rehabilitation. This questionnaire is a valid and reliable tool with construct validity and inter-rater reliability of 0.5 and 0.7, respectively (Bamm et al. 2015). The questionnaire is a self-administered questionnaire with 27 items on a 7-point Likert- response scale ranging from a score of zero (0) (‘Not applicable’) to seven (7) (‘To a very great’). The questionnaire takes about 15 min – 20 min to complete (Bamm et al. 2015). It was piloted and analysed to assess the response latency and to ensure clarity before adopting it for our study.

### Procedure for data collection

The first author visited the medical wards and stroke unit of the hospitals to identify HCPs who met the inclusion criteria. The aim of our study was explained to them and written informed consent was provided by each participant. The questionnaires were self-administered and participants were encouraged to complete and return the questionnaires as soon as possible. The average response time was 2 days. Completed questionnaires were collected by the first author and stored in a locked cabinet until the data were extracted and analysed.

### Statistical analysis

Data collected were entered and analysed in statistical package for social sciences (SPSS) version 23.0 software. Normality was assessed using the Shapiro–Wilk tests. Descriptive statistics, namely frequency and percentages were used to summarise the data. Mean age difference among the HCP across the three study sites was compared using a Kruskal–Wallis test. Associations between the distributions of HCPs, availability and access to available protocol, staff continuous education programmes, access to CT or MRI scanning onsite, duration of rehabilitation, basis for discharge and the various hospitals were assessed using Pearson’s chi-square test or Fisher’s exact test. Statistical significance was set at *p* < 0.05.

### Ethical considerations

Ethical approval was received from the Ethical and Protocol Review Committee of the School of Healthcare Sciences, University of Pretoria (Protocol No. 68/2020), Ghana Health Service Ethics Review Committee (protocol No. GHS-ERC 010/02/20) and Korle Bu Teaching Hospital Ethical and Protocol Review Committee (Protocol No. KBTH-IRB/000165/2019). Permission was sought from the heads of the hospitals and the departments where data were collected. Written informed consent was sought from each of the participants. Participants’ confidentiality and anonymity were assured. All COVID-19 safety measures were duly observed.

## Results

### Demographic characteristics of healthcare professionals

A total of 111 (*n* = 111) HCPs were included. Of these, 26% (*n* = 29) were from the primary level hospital (PH), 22% (*n* = 24) from the secondary level hospital (SH) and 52% (*n* = 58) from the tertiary level hospital (TH). The age of the participants ranged from 22 to 37 years for PH, 24 to 38 years for SH and 25 to 44 years for TH. Of the recruited participants from the TH, 40.4% (*n* = 23) were males and 59.6% (34) were females. At the SH, 39.1% (*n* = 9) of the participants were males and 60.9% (*n* = 14) were females. All participants recruited from the PH were females.

### Structure of stroke rehabilitation

**FIGURE 1:**
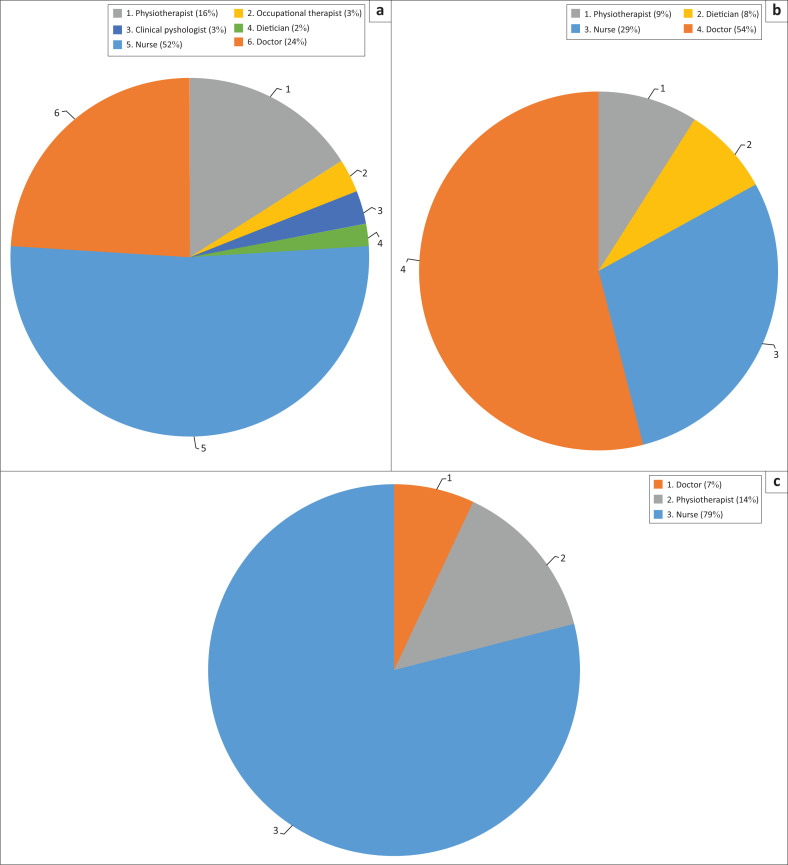
Distribution of healthcare professionals at the tertiary, secondary and primary level hospitals.

At the PH and SH, stroke patients were admitted to the general medical wards with a total number of 14 and 31 beds, respectively. However at the TH, patients were admited to a dedicated stroke unit and general medical wards with a total of 15 and 28 beds, respectively. The distribution of the HCPs was found to be influenced significantly by the level of hospital (*X*^2^ test = 26, *p* < 0.004). At the TH, the HCPs involved in stroke rehabilitation included nurses, medical personnel, physiotherapists, occupational therapists, clinical psychologists and dieticians. However, at the SH, nurses, medical personnel, physiotherapists and dieticians were the only professionals involved in stroke rehabilitation. Finally, in the PH, only nurses, medical personnel and physiotherapists were involved in stroke rehabilitation as shown in Figure 1.

**FIGURE 2:**
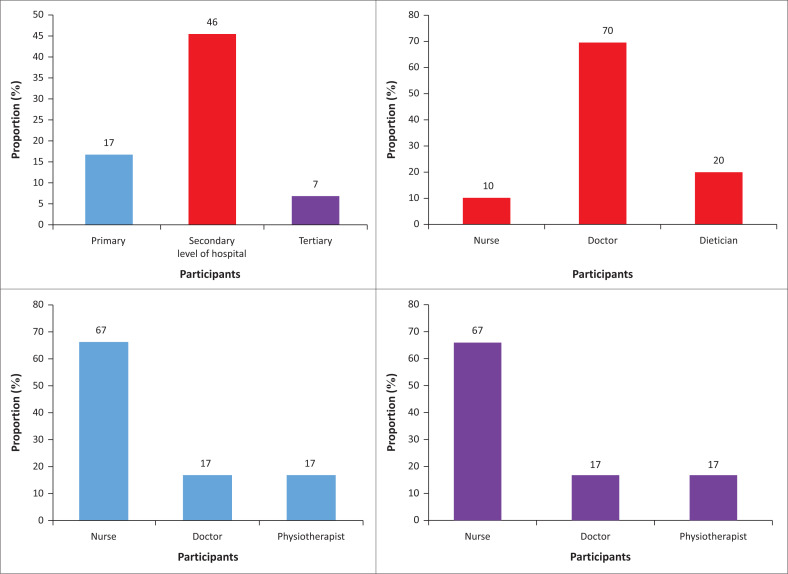
Proportion of participants reporting lack of access to stroke management protocol or guidelines.

A total of 82% of HCPs from the PH, 54% from the SH and 93% from the TH indicated that management guidelines or protocols for stroke management are available and the guidelines or protocols were readily accessible to staff. However, the level of hospital was found to significantly influence access to the available stroke management protocol or guideline, with a significant proportion of the HCPs at the SH indicating that they do not have access to the available stroke management protocol in the hospital (*X*^2^ test = 12, *p* = 0.002), as shown in Figure 2. The predominant HCPs who indicated that they do not have access to protocols were nurses, except at the SH where medical personnel contribute the greater proportion of those who do not have access to available management protocols.

Staff continuous education programmes were available at all three hospitals, and these are run weekly in the TH and PH, while at the SH, staff continuous education programmes are mostly carried out every quarter of the year.

**FIGURE 3:**
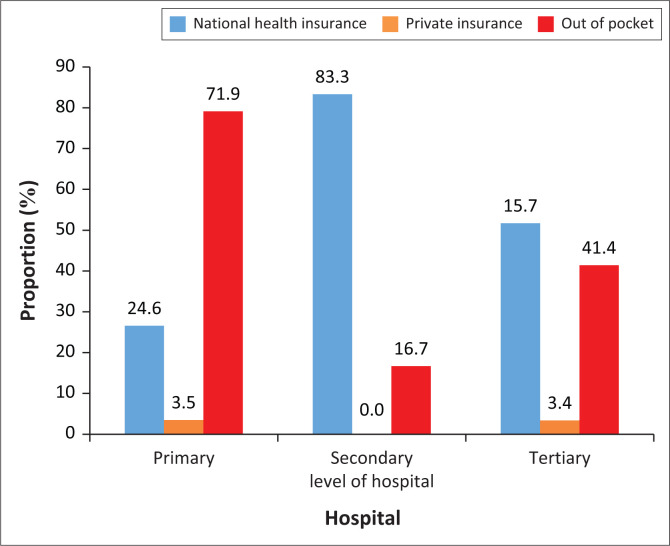
Rehabilitation payment system available at the primary-, secondary- and tertiary-level hospitals.

All patients’ access to the TH was through physician referral. Although the PH and SH are not strictly accessed through physician referral, most of the HCPs (primary = 100%, secondary = 79.2%) indicated that most of the stroke patients access the hospitals through physician’s referral. The payment system for stroke rehabilitation at all three hospitals included national health insurance, private insurance and ‘out of pocket’. However, the most used payment system for stroke rehabilitation was ‘out of pocket’ as shown in Figure 3.

The TH had more stroke rehabilitation equipment and assistive devices than the SH and PH, with the PH having the least and the rehabilitation equipment ranging from mobility aids to exercise therapy equipment and electrotherapy modalities.

### Process of stroke rehabilitation

The frequency of rehabilitation sessions, access to contrast tomography (CT) and magnetic resonance imaging (MRI) scanning on site was found to be dependent on the level of the hospital (rehabilitation sessions: *X*^2^ = 56, *p* = 0.001, CT scan: *X*^2^ = 15, *p* = 0.001, MRI: *X*^2^ = 28, *p* < 0.001). The number of rehabilitation sessions carried out by the rehabilitation team was five times a week at the TH, while it was mostly twice a week at the SH and PH. Contrast tomography (CT) and Magnetic resonance imaging (MRI) scanning on-site were readily available at the TH, while at the SH and PH CT and MRI were not readily available.

The duration of patient rehabilitation, length of hospital stay (LOS) for stroke rehabilitation and the basis of patient discharge from acute care was not found to be associated with the level of the hospital (duration of patient rehabilitation): *X*^2^ = 8.4, *p* = 0.35, (LOS): *X*^2^ = 0.94, *p* = 0.97, (basis of patient discharge) from acute care: *X*^2^ = 7.3, *p* = 0.19). Duration of patient rehabilitation was about 30 min – 40 min per session and the LOS for stroke rehabilitation was about 2–3 weeks at all three hospitals.

The basis of patient discharge from acute care was when patients were medically stable and could access outpatient management at all the hospitals. The outpatient management destinations were physiotherapy management, occupational therapy, speech and language therapy, diet therapy and clinical psychology at the TH. At the SH, the outpatient management destinations were physiotherapy management and diet therapy, while it was physiotherapy management only at the PH.

Family and patient-centredness of care in the process of stroke rehabilitation was present in all three hospitals for all four domains of family and patient-centredness of care, namely interpersonal sensitivity, provision of general information, communication of specific information and respectful treatment of patients as shown in Appendix Table 1-A1. All items under each domain were considered as part of the patient management process.

## Discussion

Our study evaluated the structure and process of stroke rehabilitation at primary, secondary and tertiary hospitals in the Greater Accra region of Ghana. According to the Ghana Health Service recommendations, all these hospitals should be involved in rehabilitation of patients with stroke. Our findings show that the structure and process of stroke rehabilitation across the different levels of care were different for the type of rehabilitation unit, constituents of the Multidisciplinary team (MDT) payment system, therapy sessions and availability of CT/MRI scanning. They were similar in protocol availability, basis of discharge, LOS and family or patient centredness of care.

Unlike the TH where acute stroke care is offered in a specialised stroke unit, the PH and SH provide stroke rehabilitation in general medical wards. However, the TH also rehabilitates patients with stroke in general medical wards. International guidelines for stroke rehabilitation and the American Stroke Association recommend that stroke rehabilitation should be carried out in organised stroke rehabilitation units as a stroke unit care provides better patient outcomes than general medical ward care (Adams et al. 2003; Langhorne et al. 2002). This system of stroke rehabilitation in stroke units has been adopted by most high-income countries recording high success rates, with patients returning to their normal function (Christian et al. 2016; Ras 2009). Although the TH evaluated here is the leading national health centre in Ghana and the third largest in Africa, with a bed capacity of about 2000, the dedicated stroke rehabilitation unit in this hospital has only 15 beds. Hence, most of the patients with stroke in this hospital are also managed in general medical wards, as they are at all levels of hospital. This, therefore, raises a concern as only a small proportion of patients with stroke in Ghana can access this designated stroke rehabilitation unit. There is, therefore, a need for the expansion of this stroke unit and the establishment of stroke units in hospitals across the nation.

Although the HCPs indicated an MDT approach in stroke rehabilitation at all three levels of hospital, the constituents of the MDT were found to be dependent on the level of hospital. The THs have diverse HCPs as compared with the PH and SH. The PH and SH have only nurses, medical personnel, physiotherapists and dieticians in the MDT in stroke care. This shows the limitations in the MDT in stroke management at PH and SH, and this suggests that patients might not receive holistic care, given that some disciplines for stroke rehabilitation are not available. This is similar to Baatiema et al. (2017a) who also report the lack of enough HCPs in stroke management in Ghana especially the allied HCPs and recommend that there should be an improvement in the constituents of MDT to optimise patient care. As a result of complications associated with post-stroke morbidity, an MDT approach of care involving nurses, medical personnel physiotherapists, occupational therapists, speech and language therapists, as well as clinical psychologists, dieticians, ophthalmologists, orthotists and psychiatrists, are recommended to be involved in stroke rehabilitation (Grube et al. 2012; Winstein et al. 2016). Patients with stroke managed by a MDT have improved outcomes and an increased likelihood of gaining functional independence in activities of daily living (Clarke 2013).

A large proportion of HCPs in the SH as compared with PH and TH indicated that although stroke rehabilitation protocols or guidelines are available, they do not have access to them. Medical personnel have the highest proportion of those who do not have access to available stroke rehabilitation protocols or guidelines in the SH, while in the PH and TH, nurses contributed to the highest proportion of those who do not have access to available stroke rehabilitation protocols. This suggests that stroke rehabilitation protocols are not well disseminated among the HCPs within hospitals and among the hospital levels. This could be attributed to the lack of a national policy for stroke management in Ghana (Baatiema et al. 2017b).

In Ghana, patients are expected to access PH and SH without being referred by a physician (Amoah & Phillips 2017). However, we found that patients with stroke access these hospitals through physician referral. Although we did not assess the source of referrals, it is possible that the patients are being referred from private hospitals or herbal centres to the government facilities. Sarfo and Ovbiagele (2021) confirm that about 50% of stroke survivors in Ghana prefer to seek management from herbal centres as there is the belief that outcomes from herbal centres are better.

The HCPs indicated the existence of a ‘cash and carry’ system of stroke rehabilitation at all three levels of hospital in Ghana where patients pay for services out of their pocket. At the PH where basic health care is expected to be available for all patients, the ‘out of pocket’ system of payment was the second most used system in stroke rehabilitation. Moreover, at the TH where a better care of stroke rehabilitation can be obtained, the most used system of payment for stroke rehabilitation is also ‘out of pocket’ This possibly contributes to the reason why most patients with stroke seek healthcare in the prayer camps and unapproved herbal centres as they are relatively cheap.

Thus, where a patient with stroke seeks healthcare can significantly influence the rehabilitation process. This is evidenced as patients in the TH receive rehabilitation 5 days per week, while patients in PH and SH receive rehabilitation 2 days per week. This suggests that stroke rehabilitation has not been prioritised in Ghana. This is illustrated by the fact that the Ghana Ministry of Health in 2012 developed a national policy for the prevention and control of chronic non-communicable diseases (Ministry of Health 2012), but information on stroke management was not made explicit in this policy despite the burden of stroke.

Although the Ghana Health Service recommends stroke rehabilitation at all the three levels of hospital, CT and MRI scanning that will aid in the rehabilitation of stroke patients are only available at the TH. This is in contrast to Baatiema et al. (2017b) who recommended that imaging services should be readily available in hospitals, especially the primary and secondary hospitals as most patients with stroke in Ghana are likely to be managed in these hospitals before they get referred to tertiary hospitals.

The LOS was 2–3 weeks, and the basis for discharge from inpatient care was when patients are medically stable and can access outpatient management. However, a longer duration of hospital stay of between 24 and 40 days (Stuart et al. 2005) show better patient outcomes, and the basis for discharge is when patients have some level of functional independence and their need of support is as minimal as possible. We show that on discharge patients only receive outpatient management but they are not followed up to their homes and communities. Other studies show that upon discharge from inpatient management, patients are not only referred to outpatient management but also receive home-based rehabilitation where patients are followed up and managed in their homes. This helps to facilitate their reintegration into their home and community (Rhoda, Mpofu & DeWeerdt 2009), contributing to improvements in functional outcomes (Aadal et al. 2018).

Family and patient-centredness of care as part of the process of stroke patient rehabilitation was present at all levels of hospital and its integration in the process of care is good. A review conducted by Jayadevappa and Chhatre (2011) found that involving patients and their family in health care helps to improve the quality of care and overall patient satisfaction with care. They also found that to achieve the best of patient outcomes, it is important to make patients and their families central to the healthcare system.

## Conclusion

Structure wise, the three levels of hospital in Ghana were found to differ in the constituents of the MDT involved in stroke rehabilitation and availability of equipment. There are no specific units dedicated to stroke rehabilitation at PH and SH as recommended by international guidelines for stroke rehabilitation. Although the TH has a dedicated unit for stroke rehabilitation, the bed capacity of the unit is only 15. Hence, most of the patients with stroke in this hospital are also managed in general medical wards. Stroke rehabilitation protocols or guidelines are not well disseminated among the HCPs within and amongst the three level of hospital. In addition, ‘out of pocket’ system of payment is the second most used system in stroke rehabilitation at the PH and the most used system of payment for stroke rehabilitation at the TH. There is, therefore, the need to increase the coverage of the National Health Insurance Scheme for acute stroke care in Ghana. The process of rehabilitation, the duration of patient rehabilitation, the LOS for stroke rehabilitation, the basis of patient discharge from acute care and family and patient centredness of care were found to be the same at all three levels of hospitals. The frequency of rehabilitation sessions was different across the three hospitals and the TH was the only hospital with access to CT and MRI scanning onsite.

We therefore recommend that policymakers in the Ghanaian healthcare system should investigate equipping the PH and SH with the needed structure as patients with stroke are rehabilitated at all levels of health care.

## Appendix 1

**TABLE 1-A1:** Patient and family centredness of care.

Item	Domains	Response	Hospitals					
	In the past year, to what extent did you		Tertiary level hospital		Secondary level hospital		Primary level hospital	
			*n*	%	*n*	%	*n*	%
**Showing interpersonal sensitivity**								
1	Suggest treatment activities with patient and family needs and lifestyle	Not at all	1	1.8	2	8.3	1	3.6
		Small extent	4	7.1	2	8.3	2	7.1
		Great extent	33	58.9	10	41.7	18	64.3
		Very great extent	18	32.1	10	41.7	7	25.0
2	Offer patient and family positive feedback or encouragement	Not at all	1	1.8	1	4.2	0	0.0
		Small extent	2	3.6	1	4.2	2	7.1
		Great extent	32	58.2	20	83.3	16	57.1
		Very great extent	20	36.4	2	8.3	10	35.7
3	Take the time to establish rapport with patients and families	Not at all	0	0.0	0	0.0	0	0.0
		Small extent	2	3.6	1	4.2	0	0.0
		Great extent	24	43.6	10	41.7	18	64.3
		Very great extent	29	52.7	13	54.2	10	35.7
4	Discuss expectations for each patient with other service providers	Not at all	1	1.8	2	8.3	1	3.6
		Small extent	6	10.9	1	4.2	3	10.7
		Great extent	27	49.1	17	70.8	19	67.9
		Very great extent	21	38.2	4	16.7	5	17.9
5	Tell patients and families about options for services or treatments for their condition	Not at all	0	0.0	0	0.0	0	0.0
		Small extent	1	1.8	0	0.0	2	7.1
		Great extent	29	52.7	17	70.8	18	64.3
		Very great extent	25	45.5	7	29.2	8	28.6
8	Discuss or explore each patient and family’s feelings about having a condition	Not at all	0	0.0	1	4.3	0	0.0
		Small extent	4	7.3	3	13.0	0	0.0
		Great extent	30	54.5	14	60.9	24	85.7
		Very great extent	21	38.2	5	21.7	4	14.3
9	Anticipate patients’ and families’ concerns by offering information even before they ask	Not at all	0	0.0	1	4.3	0	0.0
		Small extent	2	3.6	1	4.3	1	3.6
		Great extent	40	72.7	17	73.9	23	82.1
		Very great extent	13	23.6	4	17.4	4	14.3
11	Let patients and families choose when to receive information and the type of information they wanted	Not at all	2	3.7	1	4.2	0	0.0
		Small extent	8	14.8	3	12.5	3	10.7
		Great extent	31	57.4	15	62.5	22	78.6
		Very great extent	13	24.1	5	20.8	3	10.7
12	Help each family to secure a stable relationship with at least one service provider	Not at all	1	1.8	1	4.2	0	0.0
		Small extent	6	10.9	8	33.3	1	3.6
		Great extent	29	52.7	9	37.5	22	78.6
		Very great extent	19	34.5	6	25.0	5	17.9
21	Help patients and families to feel competent in managing their own care	Not at all	0	0.0	0	0.0	0	0.0
		Small extent	2	3.7	0	0.0	0	0.0
		Great extent	32	59.3	17	73.9	17	63.0
		Very great extent	20	37.0	6	26.1	10	37.0
**Providing general information**								
23	Promote family-to-family ‘connections’ for social, informational or shared experiences	Not at all	2	3.7	1	4.3	1	3.6
		Small extent	5	9.3	6	26.1	0	0.0
		Great extent	33	61.1	13	56.5	23	82.1
		Very great extent	14	25.9	3	13.0	4	14.3
24	Provide support to help families cope with the impact of the chronic condition	Not at all	0	0.0	1	4.3	1	3.6
		Small extent	6	11.3	4	17.4	1	3.6
		Great extent	32	60.4	16	69.6	19	67.9
		Very great extent	15	28.3	2	8.7	7	25.0
25	Provide advice on how to get information or to contact other patients	Not at all	0	0.0	3	14.3	2	7.4
		Small extent	10	18.9	8	38.1	1	3.7
		Great extent	35	66.0	7	33.3	9	70.4
		Very great extent	8	15.1	3	14.3	5	18.5
26	Provide opportunities for the entire family to information	Not at all	0	0.0	2	9.5	1	3.7
		Small extent	4	7.5	5	23.8	1	3.7
		Great extent	35	66.0	13	61.9	18	66.7
		Very great extent	14	26.4	1	4.8	7	25.9
27	Have general information available about different concerns	Not at all	2	3.8	1	5.0	0	0.0
		Small extent	4	7.5	5	25.0	2	7.4
		Great extent	26	49.1	13	65.0	18	66.7
		Very great extent	21	39.6	1	5.0	7	25.9
**Communicating specific information**								
14	Tell patients about the results from tests and/or assessments	Not at all	1	1.9	0	0.0	1	3.8
		Small extent	2	3.7	0	0.0	0	0.0
		Great extent	35	64.8	14	60.9	15	57.7
		Very great extent	16	29.6	9	39.1	10	38.5
15	Provide patients with written information about their condition, progress or treatment	Not at all	3	5.6	4	17.4	1	3.6
		Small extent	7	13.0	2	8.7	0	0.0
		Great extent	33	61.1	14	60.9	18	64.3
		Very great extent	11	20.4	3	13.0	9	32.1
16	Tell patients and families details about their services	Not at all	0	0.0	0	0.0	0	0.0
		Small extent	2	3.7	3	13.6	0	0.0
		Great extent	33	61.1	13	59.1	19	67.9
		Very great extent	19	35.2	6	27.3	9	32.1
**Treating people respectfully**								
6	Accept patients and their family in a non-judgemental way	Not at all	0	0.0	0	0.0	0	0.0
		Small extent	0	0.0	1	4.2	1	3.6
		Great extent	31	56.4	13	54.2	13	46.4
		Very great extent	24	43.6	10	41.7	14	50.0
7	Trust patients as the ‘experts’ on themselves	Not at all	1	2.0	5	23.8	1	4.2
		Small extent	7	14.3	3	14.3	2	8.3
		Great extent	30	61.2	9	42.9	16	66.7
		Very great extent	11	22.4	4	19.0	5	20.8
10	Make sure patients and families had a chance to say what was important to them	Not at all	0	0.0	0	0.0	0	0.0
		Small extent	1	1.8	1	4.2	1	3.6
		Great extent	28	50.9	13	54.2	23	82.1
		Very great extent	26	47.3	10	41.7	4	14.3
13	Answer patients’ and families’ questions completely	Not at all	0	0.0	0	0.0	0	0.0
		Small extent	2	3.6	1	4.2	0	0.0
		Great extent	22	40.0	15	62.5	16	57.1
		Very great extent	31	56.4	8	33.3	12	42.9
17	Treat each patient and their family as an individual rather than as a ‘typical’ patient	Not at all	0	0.0	1	4.5	0	0.0
		Small extent	1	1.9	1	4.5	0	0.0
		Great extent	36	67.9	12	54.5	19	67.9
		Very great extent	16	30.2	8	36.4	9	32.1
18	Treat patients as equals rather than just as a patient	Not at all	1	1.9	0	0.0	1	3.7
		Small extent	3	5.7	1	4.5	1	3.7
		Great extent	28	52.8	11	50.0	15	55.6
		Very great extent	21	39.6	10	45.5	10	37.0
19	Make sure patients and families had opportunities to explain their treatment goals and needs	Not at all	0	0.0	1	4.3	0	0.0
		Small extent	2	3.8	0	0.0	0	0.0
		Great extent	32	60.4	14	60.9	19	67.9
		Very great extent	19	35.8	8	34.8	9	32.1
20	Help patients and families feel like a partner in their own care	Not at all	0	0.0	0	0.0	0	0.0
		Small extent	2	3.7	1	4.3	1	3.6
		Great extent	32	59.3	15	65.2	18	64.3
		Very great extent	20	37.0	7	30.4	9	32.1
22	Treat patients and their families as people rather than as a case	Not at all	1	1.9	1	4.3	0	0.0
		Small extent	4	7.4	0	0.0	1	3.6
		Great extent	26	48.1	17	73.9	17	60.7
		Very great extent	23	42.6	5	21.7	10	35.7
	Average	Not at all	0	0.0	1	4.2	0	0.0
		Small extent	2	3.6	2	8.3	0	0.0
		Great extent	39	70.9	18	75.0	22	78.6
		Very great extent	14	25.5	3	12.5	6	21.4
